# Medical Education in the State of New York

**Published:** 1883-10

**Authors:** 


### Editorial.

### Medical Education in the State of New York.

Ever since the Journal has been under its present editorial management it has been outspoken in exposing the iniquities of our present system of medical education; and we have urged with all boldness the application of the only effective remedy, namely, the complete separation of the unfortunate, unwise and debasing copartnership of the educating and licensing powers. As a brave step in the right direction, therefore, we rejoice to be able to call the attention of our readers to the proceedings of the Erie County Medical Society, as published in this issue. The committee on legislation have well done the duty imposed upon them, and deserve the thanks and hearty support of every member of the profession in the State of New York.

The subject of State control of the practice of medicine in other commonwealths has been well studied, the peculiarities of our own State regulations and institutions critically observed, and a bill drafted which seeks to embody the necessities and advantages of both. More than this, the committee appointed to do this work, by a bare majority vote, have so won the confidence of the profession that the draft of the bill reported by them, after careful consideration and thorough discussion, was unanimously adopted. We consider this latter fact of the most hopeful augury.

As will be seen, the bill is by no means a compromise. Far from it. On every issue involved in this question there is the most unmistakably plain speaking.

The body which this bill would create is a "mixed" body, will be the sole licensing body in and for this State, and will not be composed of, nor controlled by, the professors of our too many medical schools.

More than this, this faculty, the sole representative medical body in the State, will have the power to say what medical schools in this and other countries are in good standing in this State, and also power to declare what is and what is not professional conduct. Surely these are not compromise provisions; and that a full meeting of our county society could endorse such a plan convinces us that our whole profession is seriously in earnest in this business, and willing, in many instances, to drop individual preferences, rather than risk the chance of co-operating on the main question.

As we have before said, this is "something worth fighting for," and it is an augury of success that all the great medical journals of the State are speaking with no uncertain sound in support of the bill. The interest in the success of the bill is by no means confined to the State, but the movement is watched with intense interest by the profession all over the country, and a victory in the battle for higher medical education in the Empire State, ensures the rapid introduction of similar laws in every State of the Union. The *Philadelphia Medical Times*, among others, speaks out boldly on the subject, and urges, as the sole remedy for the present iniquitous system of medical education, the taking from the colleges the right to grant licenses to practice. "So long as men can make thousands of dollars yearly by the sale of diplomas, and hide their crime under the cloak of teaching, so long will the consciences of many be dumb and without feeling." "Whether we like it or not," says the *Times*, "if success is to be achieved, the existence of homœopathy must be recognized in the formation of the board." This opinion is concurred in by every one who has given the subject serious thought.

And now to work. The matter must not be left to officers of societies. We call upon every individual member of the profession to use all his influence to secure the passage of the bill. The committee propose to place a copy of it in the hands of every medical man in the State, and if physicians do their duty, every member of the coming legislature can be made to understand all the provisions of the bill, to recognize their fairness and necessity, and to be prepared to vote in favor of it.

